# The Ontology for Biomedical Investigations

**DOI:** 10.1371/journal.pone.0154556

**Published:** 2016-04-29

**Authors:** Anita Bandrowski, Ryan Brinkman, Mathias Brochhausen, Matthew H. Brush, Bill Bug, Marcus C. Chibucos, Kevin Clancy, Mélanie Courtot, Dirk Derom, Michel Dumontier, Liju Fan, Jennifer Fostel, Gilberto Fragoso, Frank Gibson, Alejandra Gonzalez-Beltran, Melissa A. Haendel, Yongqun He, Mervi Heiskanen, Tina Hernandez-Boussard, Mark Jensen, Yu Lin, Allyson L. Lister, Phillip Lord, James Malone, Elisabetta Manduchi, Monnie McGee, Norman Morrison, James A. Overton, Helen Parkinson, Bjoern Peters, Philippe Rocca-Serra, Alan Ruttenberg, Susanna-Assunta Sansone, Richard H. Scheuermann, Daniel Schober, Barry Smith, Larisa N. Soldatova, Christian J. Stoeckert, Chris F. Taylor, Carlo Torniai, Jessica A. Turner, Randi Vita, Patricia L. Whetzel, Jie Zheng

**Affiliations:** 1 University of California San Diego, La Jolla, California, United States of America; 2 British Columbia Cancer Research Centre, Vancouver, British Columbia, Canada; 3 University of Arkansas for Medical Sciences, Little Rock, Arkansas, United States of America; 4 Oregon Health and Science University, Portland, Oregon, United States of America; 5 Drexel University College of Medicine, Philadelphia, Pennsylvania, United States of America; 6 University of Maryland School of Medicine, Baltimore, Maryland, United States of America; 7 Thermo Fisher Scientific, Carlsbad, California, United States of America; 8 Simon Fraser University, Burnaby, British Columbia, Canada; 9 The Vrije Universiteit Brussel, Ixelles, Brussels, Belgium; 10 Stanford University, Stanford, California, United States of America; 11 Ontology Workshop, LLC, Columbia, Maryland, United States of America; 12 National Toxicology Program, NIEHS, National Institutes of Health, Research Triangle Park, North Carolina, United States of America; 13 Center for Biomedical Informatics and Information Technology, National Institutes of Health, Rockville, Maryland, United States of America; 14 Royal Society of Chemistry, Cambridge, Cambridgeshire, United Kingdom; 15 University of Oxford, Oxford, Oxfordshire, United Kingdom; 16 University of Michigan Medical School, Ann Arbor, Michigan, United States of America; 17 National Cancer Institute, Rockville, Maryland, United States of America; 18 University at Buffalo, Buffalo, New York, United States of America; 19 Newcastle University, Newcastle-upon-Tyne, Tyne and Wear, United Kingdom; 20 European Molecular Biology Laboratory- European Bioinformatics Institute, Hinxton, Cambridgeshire, United Kingdom; 21 University of Pennsylvania, Philadelphia, Pennsylvania, United States of America; 22 Southern Methodist University, Dallas, Texas, United States of America; 23 The University of Manchester, Manchester, Greater Manchester, United Kingdom; 24 La Jolla Institute for Allergy and Immunology, La Jolla, California, United States of America; 25 J. Craig Venter Institute, La Jolla, California, United States of America; 26 Leibniz Institute of Plant Biochemistry, Halle, Saxony-Anhalt, Germany; 27 Brunel University London, Uxbridge, Middlesex, United Kingdom; 28 Georgia State University, Atlanta, Georgia, United States of America; Huazhong University of Science and Technology, CHINA

## Abstract

The Ontology for Biomedical Investigations (OBI) is an ontology that provides terms with precisely defined meanings to describe all aspects of how investigations in the biological and medical domains are conducted. OBI re-uses ontologies that provide a representation of biomedical knowledge from the Open Biological and Biomedical Ontologies (OBO) project and adds the ability to describe how this knowledge was derived. We here describe the state of OBI and several applications that are using it, such as adding semantic expressivity to existing databases, building data entry forms, and enabling interoperability between knowledge resources. OBI covers all phases of the investigation process, such as planning, execution and reporting. It represents information and material entities that participate in these processes, as well as roles and functions. Prior to OBI, it was not possible to use a single internally consistent resource that could be applied to multiple types of experiments for these applications. OBI has made this possible by creating terms for entities involved in biological and medical investigations and by importing parts of other biomedical ontologies such as GO, Chemical Entities of Biological Interest (ChEBI) and Phenotype Attribute and Trait Ontology (PATO) without altering their meaning. OBI is being used in a wide range of projects covering genomics, multi-omics, immunology, and catalogs of services. OBI has also spawned other ontologies (Information Artifact Ontology) and methods for importing parts of ontologies (Minimum information to reference an external ontology term (MIREOT)). The OBI project is an open cross-disciplinary collaborative effort, encompassing multiple research communities from around the globe. To date, OBI has created 2366 classes and 40 relations along with textual and formal definitions. The OBI Consortium maintains a web resource (http://obi-ontology.org) providing details on the people, policies, and issues being addressed in association with OBI. The current release of OBI is available at http://purl.obolibrary.org/obo/obi.owl.

## Introduction

Information derived from biomedical investigations is increasingly being captured in structured electronic formats and made available through public database resources as a complement to reporting in traditional journal publications. For many years, different formats to describe experiments have been developed in isolation, as each research community tended to focus on a specific methodology[1, 2]. The ‘many communities, many standards’ approach supported the needs of individual communities but resulted in common features of different investigations being described in different ways. This situation created difficulties for representing cross-disciplinary experiments, for example, integrative meta-analysis spanning multiple scientific communities[3, 4]. To support interoperability between different database systems, the key features of an investigation need to be described in a common, shared and unambiguous manner. Minimally, this requires standards for the representation of samples, assays and data analysis methods used in an investigation. It is this background that provides the motivation for the Ontology for Biomedical Investigations (OBI; http://purl.obolibrary.org/obo/obi.owl).

OBI addresses the requirement for a cross-disciplinary standard for representing biomedical investigations. It is both broad, describing the parts of an investigation from conception to conclusion, and deep, describing entities from test tubes to transgenic organisms. As an ontology, OBI is a controlled vocabulary with additional logical constraints, reusing basic terms to build more complex ones. OBI is expressed in the OWL 2 Web Ontology Language (http://www.w3.org/TR/owl2-overview/), which is a World Wide Web Consortium (W3C; http://www.w3.org) ontology language developed for the semantic web (see Methodology). It supports and augments existing community standards such as Microarray Gene Expression tab-delimited format (MAGE-TAB)[5], Functional Genomics Experiment (FuGE)[1] and Investigation, Study, Assay tab-delimited format (ISA-Tab)[4].

OBI is publicly and freely available under the Creative Commons Attribution (CC-by) 3.0 license. It is a community based, globally distributed grass-roots project in the tradition of many standardization efforts that have joined forces in this endeavor[6–9]. It has representatives from many disciplines; covering much of biomedicine and including informatics expertise (see S1 Table for a list of contributing projects). The consortium has an open membership policy and a transparent development process. OBI development is driven by the needs of its contributing members and by outside user requests. New terms are typically added to enable describing specific examples of experiments which are not yet covered in OBI, and then generalizing them. As such, OBI is not complete—and never will be—but rather will continue to evolve as new examples are introduced.

The primary use of ontologies in bioinformatics has been the annotation of database records. OBI supports and is presently being used for this task. In addition, OBI has a rich underpinning of computational logic; this can support the annotation process by automatically checking for consistency, as well as enabling rich querying over data repositories. Being built on W3C standards, it can also use and be used with other data available on the semantic web (see Methodology).

OBI follows and has helped to form, the framework set out by the OBO Foundry[10]. In 2013 OBI was reviewed by an OBO Foundry committee and is now one of eight ontologies with full OBO Foundry membership status. OBO Foundry membership indicates that OBI was found to sufficiently follow the Foundry principles. Membership status also indicates that OBI should be used by other OBO ontologies as the preferred source of terms in its domain in order to further facilitate re-use.

In this paper, we describe the current state of OBI and its usage. We outline the design decisions followed by the OBI consortium, which has enabled this development. We give an overview of the organization of the ontology and detail some of the classes and relations that form the structure of OBI. We also describe three applications which exemplify the different ways in which OBI can be used.

Further enhancements of OBI in terms of breadth and depth of coverage are driven by the participating consortium members; we welcome and encourage broader community participation in this open, collaborative ontology development effort.

## Methodology

OBI development is coordinated in weekly teleconferences in addition to occasional face-to-face meetings. Developers are dispersed globally. As a result, OBI has adopted a specific methodology and many formal conventions, to manage what would otherwise be a chaotic process. This has been critical in ensuring that OBI is a homogeneous resource. We describe these conventions here.

### Choice of metadata conventions

OBI developed and has consistently used a convention for information that documents classes (metadata). This takes the form of a number of documented annotation properties and fillers. Initially this metadata included basic information such as labels, synonyms, definitions, attribution to editors and source for definitions. As OBI’s development progressed additional properties were added as need was recognized. Curation status gives an indication of the level of development of a term, and its possible values have been augmented as different cases emerged. Various forms of additional documentation properties have been added in response to needs of developers and users. These now include example of usage of a term, notes that aid the user in understanding the term, and notes that relate to the development process. The metadata scheme is implemented as an OWL ontology available at [http://purl.obolibrary.org/obo/ontology-metadata.owl]. A specification of what is considered minimally acceptable use is available at [http://purl.obolibrary.org/obo/obi/policy/metadata]. This metadata scheme has now been adopted by a number of OBO ontologies and continues to evolve with participation from other members of the OBO community[11]. The OBI metadata specification includes definitions of the curation status values (example shown in Table 1). Their choice is reflective of the discussion about the term occurring amongst OBI developers. For example, terms that have been discussed on an OBI developers call and agreed upon for inclusion are ‘ready for release’. Terms that have been added following an agreed upon pattern but not reviewed by anyone but the term editor are ‘pending final vetting’.

**Table 1:** Example metadata for class OBI_0000795.

Annotation property	Example	Usage
Editor Preferred Term*	glucometer	The concise, meaningful, and human-readable name for a class or property that reflects community usage, or disambiguates the term
Definition*	A measurement device with the function to measure and record the level/amount of glucose in a blood sample	The Aristotelian definition, explaining the meaning of a class or property
Term Editor*	PERSON:Frank Gibson	The editors that created the term. In addition to ‘PERSON:’, ‘GROUP’: attributions are allowed. This syntax is an attempt at allowing future tools to track contributions of editors across ontologies.
Definition Source*	http://en.wikipedia.org/wiki/Glucose_meter	A traceable reference to the source of the definition.
Curation Status*	Ready for release	The curation status of a class or property, one of: uncurated; metadata incomplete; metadata complete; pending final vetting; ready for release; placeholder
Example of usage	Diabetic patients use glucometers to determine their glucose levels	A phrase describing how the term is used
Alternative Term	glucose meter	A synonym for the class or property

* required metadata

### Use of the Web Ontology Language (OWL)

OBI is developed using the OWL 2 Web Ontology Language (http://www.w3.org/TR/owl2-overview/) as this provides richer semantic support than OBO format (http://www.geneontology.org/GO.format.obo-1_2.shtml)—the other commonly used alternative in the biomedical domain. The metadata scheme is implemented as OWL annotation properties. The OWL 2 Web Ontology Language (http://www.w3.org/TR/owl2-overview/) is a W3C standard for the representation of ontologies within the larger framework of the semantic web. OWL builds on the Resource Description Framework (RDF; http://www.w3.org/TR/rdf-primer/) standard in which data is represented by sets of subject-predicate-object statements (“triples”) that form a directed graph. Subjects and predicates are named using Internationalized Resource Identifiers (IRIs; https://tools.ietf.org/html/rfc3987), while the object position can be filled by an IRI or a literal value (e.g. string or number). The XML Schema (http://www.w3.org/XML/Schema) standard helps to give structure to literal data types in RDF. RDF graphs can be queried using the SPARQL Protocol and RDF Query Language (SPARQL; http://www.w3.org/TR/sparql11-query/) which is the equivalent of Structured Query Language (SQL) for querying relational databases.

The RDF Schema (RDFS; http://www.w3.org/TR/rdf-schema/) standard provides additional tools for working with controlled vocabularies and ontologies. OWL builds on RDFS, providing a more expressive language for describing classes, individual members of those classes, “object properties” that link pairs of individuals, “data properties” that link individuals to literal values, and “annotation properties” for describing any of these. The theoretical foundation for OWL is description logic, a decidable fragment of first-order logic. Informally, “decidable” means that any question framed within this logic can be answered within a finite number of steps, unlike full first-order logic. In practice there are always limits on computational resources and time, but automated OWL reasoners will check an ontology for logical consistency and satisfiability, and draw inferences that can go well beyond the assertions made by the ontology authors.

A number of software tools support the use of OWL and were utilized in the development of OBI. These include the comprehensive OWL Application Program Interface (API) Java library[12] and the Protégé-OWL (http://protege.stanford.edu/overview/protege-owl.html) graphical editor that builds upon it. A number of OWL reasoners are available and have been tested with OBI, including HermiT[13], ELK[14], and Pellet[15], each with different capabilities.

### Concurrent development

Concurrent development of OBI was necessary given the number of developers. A review of the existing collaborative ontology development tools failed to identify a single application that met OBI’s requirements. Originally, OBI development was, therefore, organized into branches, with each sub-team working independently on a specific subject area, such as data transformations or roles. With the increasing maturity of OBI, and during the move to the 1.0 release, there was more stability and we adopted a single development line. For change management, we used the industry standard Subversion tool; the current development version of OBI is available at http://purl.obolibrary.org/obo/obi/repository.

### Integration with existing ontologies

OBI was developed to be complementary to, and integrated with, a framework of existing ontologies in the biomedical domain. For example, when an investigation involves a subject organism we include the National Center for Biotechnology Information (NCBI) Taxonomy term for its species or strain, and when an assay measures a biological process we include the Gene Ontology term for that process. Whenever possible, we reuse existing terms rather than creating our own, allowing our users to take advantage of data and annotations that already use those existing terms. This integration is more than simple linking: we incorporate external ontology terms into our definitions, logical axioms, and annotations, to create a larger framework that facilitates data integration and reuse across biology and biomedicine. The methodological challenges associated with this reuse are discussed here.

A top level ontology can provide guidance in how individual terms or whole ontologies interrelate and is useful when integrating external ontologies. Basic Formal Ontology (BFO)[16] was chosen as the top level ontology as it is stable, publicly available in OWL syntax, and aligned to existing OBO Foundry ontologies, and because there is a community of developers and users. The OBO Relations Ontology[17] provides relations for OBI. When new relations have been defined between classes in OBI, these are based on RO relations wherever possible.

The Information Artifact Ontology developed out of OBI and the two ontologies continue to have a close relationship. OBI imports all IAO terms using the OWL import mechanism. This import mechanism was not suitable for all external ontologies for two reasons: first, current editing tools are not effective for working with very large ontologies such as the NCBI Taxonomy [18], therefore a direct import is not scalable; second, some ontologies used by OBI are actively developed and may not be aligned with OBI methodology, for example they may not use BFO or OWL Description Logic (DL). Importing such ontologies as a whole can lead to inconsistencies or unintended inferences. Instead, the MIREOT import mechanism was developed[19]. MIREOT allows specification of a set of terms to be imported including mapping of metadata, placement of imported terms within the OBI hierarchy and inclusion of selected axioms. We use OntoFox [20]], which acts on these specifications, to facilitate the import of 433 terms from external ontologies into OBI with the MIREOT technique. OntoFox is re-used for each release ensuring that imported terms are kept up-to-date with their source.

### Scope limitation to canonical investigations

The scope of OBI includes the representation of successfully completed investigations, which is a type of *planned process*. A successful *investigation* may produce negative results, but processes that result in counterfeit data or during which there were gross errors are out of scope for OBI. For example, if a *PCR-SSCP assay* fails to detect a sequence variation, it is in scope for OBI. However, if controls performed as part of the assay fail then it is not in scope. We are representing only experiments which have succeeded by their own criteria, to avoid unnecessary complexity in all definitions. This is similar to the decision of the GO Consortium to model only canonical biological processes [21].

### Term requests

To add new terms to OBI, developers and external users are encouraged to submit a term request through a public term tracker (https://sourceforge.net/p/obi/obi-terms/). Submitted terms are then assessed to determine if they are in the scope of OBI. For example, terms like ‘peptide’ and ‘antibody’ are needed to describe immunological experiments, but were available in ChEBI[22] and GO respectively, and were therefore imported. Submitted terms were distributed to an appropriate OBI developer for curation and inclusion in the OWL file, and interaction of developers was achieved through conference calls and workshops.

### Release and quality control

Users require a traceable, static version of OBI. We have therefore established release process where multiple OWL files are merged into a single file. The release version of the OBI is reasoned over and contains the fully inferred hierarchy, making it easier to use and view in available tools. Each release is available from a specific URI using the release date as a tag. The most up-to-date file is always available from http://purl.obolibary.org/obo/obi.owl. Releases of OBI are also uploaded to the National Center for Biomedical Ontology (NCBO) BioPortal[23] and the Ontobee repository at http://www.ontobee.org/

Checks are made prior to release to ensure compliance with OBI policies. Specifically, classes are identified that: do not have all required metadata information (summarized in Table 1); have invalid OWL syntax; or, lead to inconsistency when reasoning using Pellet[15] or Hermit[13]. Identifier format and deprecation policies are also enforced as follows: New classes are automatically assigned a permanent and unique identifier on their first release. We are implementing checks to verify that all IDs present in the previous release are still in use as part of the release process. This follows the GO deprecation policy that OBI has adopted: deleted classes are moved to the *ObsoleteClass* in the OBI hierarchy and have the OWL *deprecated* annotation set to “true”.

## Results

### OBI classes and relations

**Fig 1:**
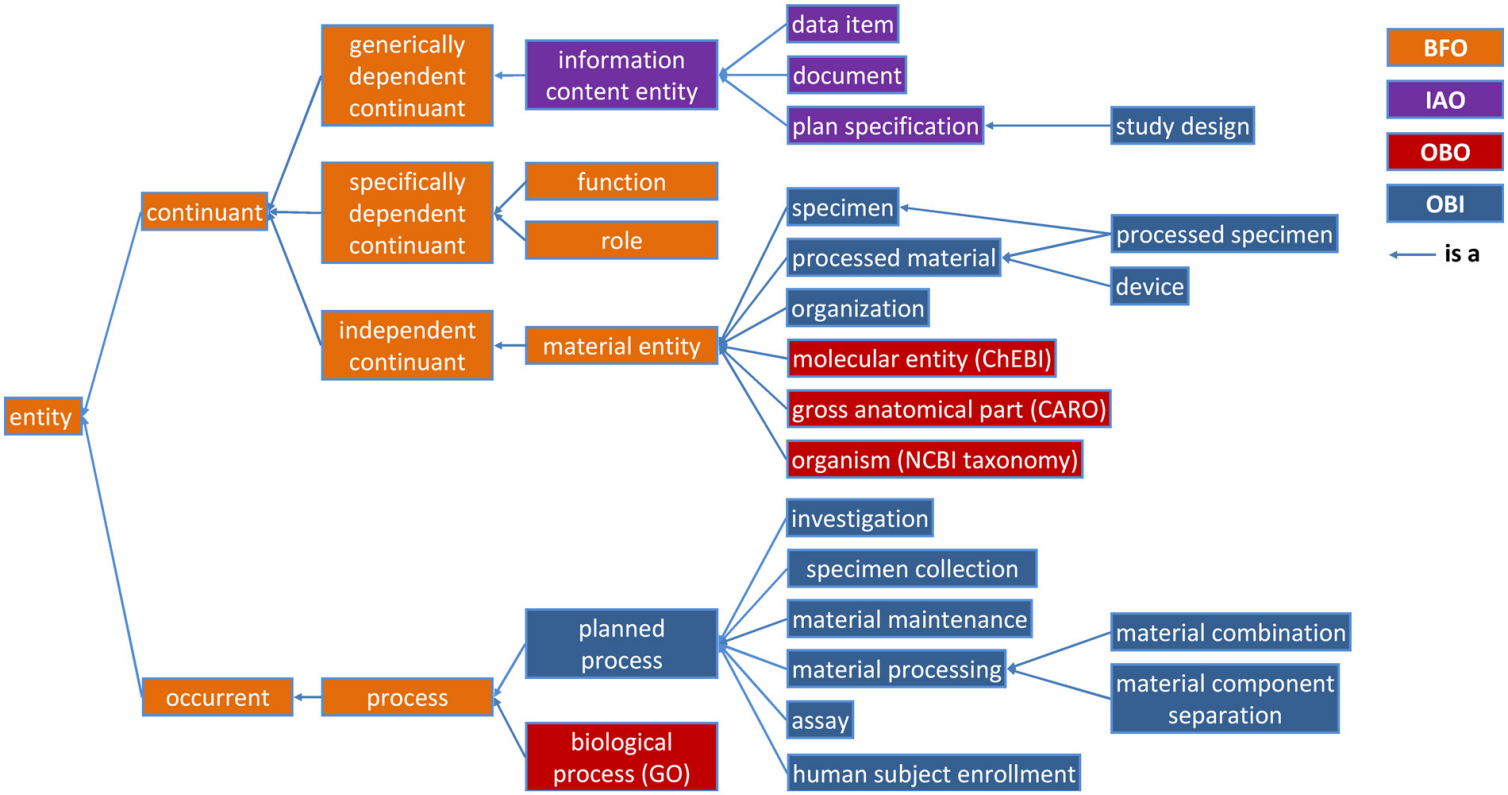
Partial high-level structure of OBI classes. OBI classes are shown in blue. Classes imported from BFO, IAO and other external ontologies are shown in orange, purple and dark red, respectively. Some example subclasses, such as *device* and *processed specimen* are included to illustrate the use of the class *processed material*.

The OBI 2015-10-20 release includes 2366 classes, 84 individuals, and 40 relations (owl:objectProperties) native to OBI plus 556 classes, 94 individuals, and 59 relations imported from 20 ontologies and the Relations Ontology (RO). The high level class organization of OBI is depicted in Fig 1. The upper level consists of the BFO classes *material entity*, *process*, *role* and *function*, and the IAO class *information content entity*. Throughout this text, *italics* are used to indicate a term denoting a class, instance or relation in an ontology. The plural of the class label is sometimes used in the text for readability, but the official OBI class labels are singular. This section gives an overview of several higher level OBI classes that outline its scope and illustrate the modeling approach.

#### Material entity

Within BFO, entities made up of matter are represented using the BFO classes of *object*, *fiat object* and *object aggregate*. The use of these BFO classes is appropriate if all entities are of similar scale. However, entities in biomedical investigations span sizes from molecules to populations of organisms. Depending on the level of granularity chosen, these might be considered unitary objects or aggregates of smaller parts. Therefore, the class *material entity* was created in BFO at the request of OBI developers as the union of *object*, *fiat object part* and *object aggregate*. By using *material entity* as our root class we avoid committing to the granularity schema in BFO.

Several subclasses of *material entity* are imported from external ontologies: Classes under *organism* are imported from the NCBI Taxonomy[18], *gross anatomical part* and its subclasses from the Uber anatomy ontology (UBERON)[24] or the Common Anatomy Reference Ontology (CARO)[25], and the *molecular entity* hierarchy from ChEBI[22]. OBI-specific classes using these imports were then constructed. For example, a *chemical solution* such as a *PBS buffer* can be defined by referencing the molecules defined in ChEBI from which the buffer is made.

Material entities that result from intentional acts are *processed material*. For example, a *blood plasma specimen* collected in a clinical protocol is a *processed material*, as is a *measurement device*, such as a *glucose meter*, which is manufactured with the intent to perform a *function*. Table 1 gives the complete metadata describing the *glucometer* class as an example. A *material entity* such as a *protein* can be produced intentionally, for example using a recombinant expression system, or naturally expressed in an organism. Class hierarchies with multiple inheritance can become difficult to maintain; therefore, *processed material* is logically defined with necessary and sufficient conditions, as exactly those material entities that *are specified output of* a process of *material processing*. This allows automated reasoners to infer subclasses. For example *peptide construct* (which is asserted as a subclass of *material entity*) is inferred by the reasoner to be a subclass of *processed material*, as it *is specified output* of some *enzymatic ligation*.

#### Planned Processes

The OBI definition of *material processing* is a type of *planned process*. Such *processes* are initiated by an agent (typically a person) to achieve a certain goal. OBI captures such goals by defining an *objective specification*, which is a subclass of *information content entity*. An example *objective specification* is the phrase “Hybridize RNA to chip” in a manuscript describing a microarray gene expression protocol[26]. When a *process* is performed to meet an *objective specification* and it is successfully completed, it is a *planned process*. The majority of processes represented in OBI are *planned processes*. OBI uses the relation *achieves planned objective* between a *planned process* and a corresponding *objective specification*. A single *planned process* can achieve more than one objective. The *planned process* hierarchy of OBI represents realizations of broad objectives. These include *material component separation*, where, for example, a cell sorter is used to separate T cells from other cells in a blood specimen, and *material combination*, such as when a chemical is added to a cell culture.

**Fig 2:**
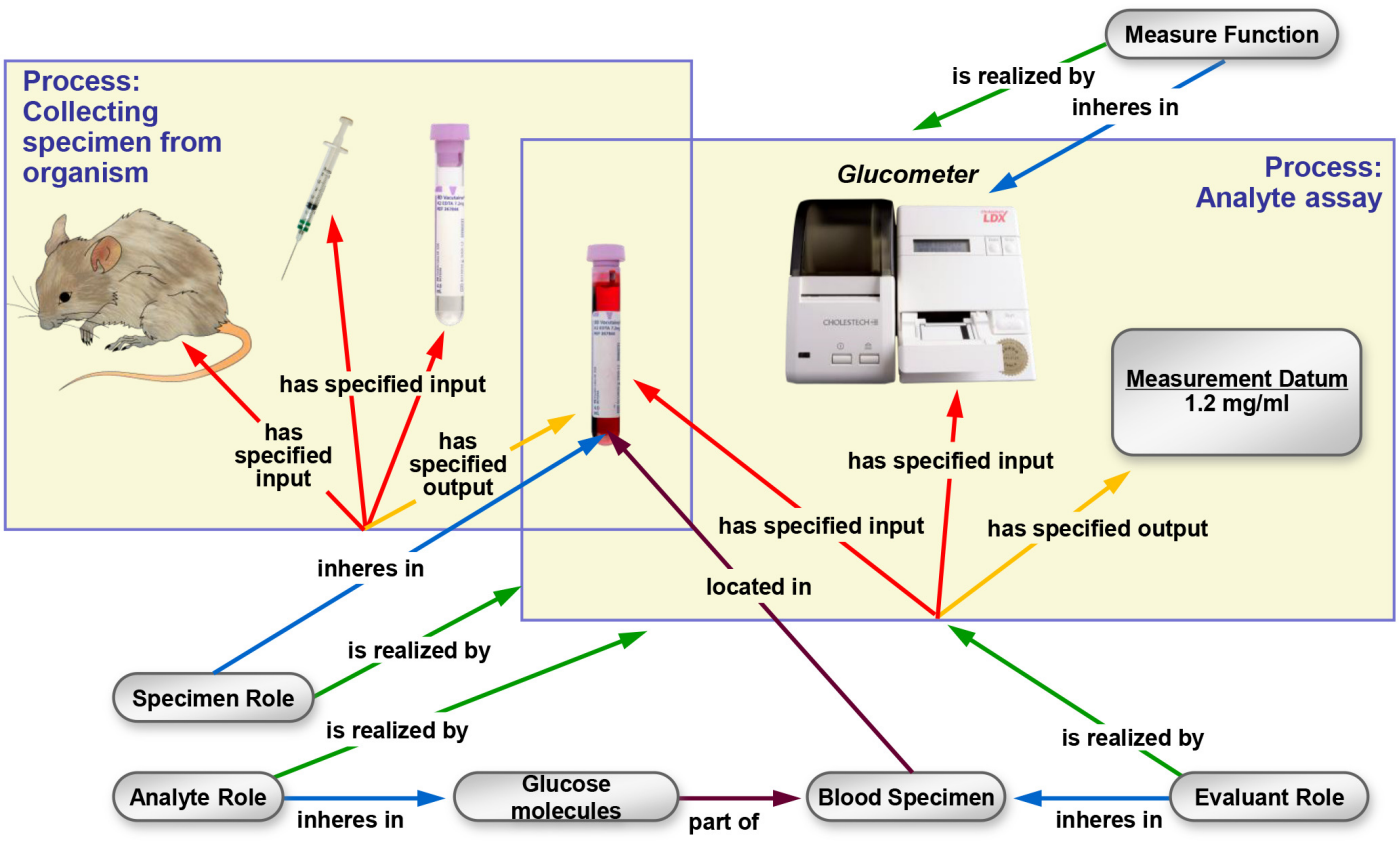
Measuring glucose concentration in blood. The large boxes represent instances of processes and their participants. The *collecting specimen from organism* process takes place first. In this process, a *syringe* is used to draw blood from the mouse. At the end of this process a tube contains the *blood specimen*. In a second process, this specimen is used in an *analyte assay*, which measures the concentration of glucose in the blood. A *glucometer* is used to make this measurement. The *analyte role* inheres in the *glucose molecules* scattered throughout the *blood specimen*. This *planned process* achieves the *analyte measurement objective*.

While processes can have many participants, *objective-* and *plan specifications* explicitly identify important participants. For example, while the light source used to illuminate a laboratory is not normally specified in a mass spectrometry experiment, the use of a mass spectrometer always is. OBI defines two relations, *has specified input* and *has specified output*, relating planned process instances to participants that are explicitly identified in their plan specification. Specified outputs must be present at the end of a process for it to achieve its objective specification. Specified inputs are identified in the *plan specification* and are not created during the process. Fig 2 gives an example of how two planned processes, drawing blood and measuring its glucose concentration, are represented. OBI also includes processes such as *data transformation*, which have *data items*, as opposed to *material entities*, as the input and output. For example, the OBI class *mean centering* achieves a *data normalization objective*.

#### Information entities

Any ontology of biomedical investigations needs to represent information such as data, investigation designs and reports of investigation results. The Information Artifact Ontology (IAO[19]) grew out of OBI efforts to capture these entities. IAO was created to provide a higher-level general ontology in recognition of the need to capture information outside of the scope of OBI (e.g., law-related). The root class of IAO is *information content entity*, which describes all information that is intentionally created. All *information content entities* share two features; they are generically dependent, so they must be borne by at least one other entity; and, that they *are about* other entities. The *is about* relationship does not specify the structure or format of the information; rather, it specifies the entity the information itself describes. So, the glucose concentration measurement in Fig 2 *is about* a *molecular concentration* quality of the specimen.

For OBI, an important subclass of *information content entity* is *directive information entity*, such as a *plan specification* or an *objective specification*, which an experimenter would read to learn how or why to perform a certain experiment. IAO also defines *documents* such as *journal articles* or *patents*, and their parts such as a *table*, *figure* or *scatter plot*.

OBI then defines subclasses of *information content entity* that are specific to investigations. These include *protocol* and *study design*, which are types of *plan specification*, representing the specification of a single procedure or an entire investigation, respectively. Many *study designs* identify as key components the *study design independent variables*, which can specify that the concentration of a chemical will be varied in otherwise identical cell cultures, and *study design dependent variables*, such as that the proliferation rate in the cell culture will be measured as a function of the chemical concentration. Central to investigations are the types of data generated. The output of an *assay* is typically a *data item*. Finally, the interpretation of information generated during the *study design execution* is documented as a *conclusion based on data*.

#### Roles and Functions

A *role* as used in OBI and defined in BFO has two properties: first, the entity that bears the *role*, and second the process in which the *role* is *realized*. A role is not essential to the entity bearing that role, but is demonstrated under certain circumstances when the role is realized. For example, humans can bear specific roles relating to investigations including the *investigation agent role*, realized by contributing to the completion of an investigation, and the *author role*, realized by writing a *report*. Additionally, OBI contains roles defined by the study design of an investigation. The results of a study can be about any material entity such as humans, tissue or molecules, and these materials studied bear a *participant under investigation role*. OBI also contains roles defined in specific experimental procedures, such as the *analyte role* or *reagent role*.

Functions differ from roles in BFO in that they are intrinsic to an entity because of structural organization[27]. For example, OBI defines that a *polystyrene tube* and an *animal cage* bear the *contain function*, while a *measure function* inheres in a *gamma counter*.

#### Organization

OBI needs to represent *organizations* to identify, for example, *Affymetrix* as the *manufacturer* of a *microarray*. Placing organization into the BFO hierarchy proved controversial, as good arguments were made for treating it either as a material entity or an immaterial kind of social construct related to other legal entities. The latter are not currently well described in BFO. In fact, both exist, though in English the same word (organization) is fluidly used to describe one or the other. At any time, an organization is comprised of a number of persons. That aspect can be captured as a material entity–the aggregate of those people (instances of homo sapiens, which are material entities). Organizations can also have other organizations as members and since those are also material entities, the whole is a material entity. Since material entities are better developed in BFO, we associated the label ‘organization’ with the material entity. The social aspects of the organization can be captured by organization-specific member roles, for example the role treasurer, or administrator, or compliance officer, although OBI has not found it necessary to define such roles. The current definition of organization is broad enough to include for example a *regulatory agency* like the *U*.*S*. *Food and Drug Administration*.

OBI needs to represent *organizations* to identify, for example, *Affymetrix* as the *manufacturer* of a *microarray*. Placing *organization* into the BFO hierarchy proved controversial, as good arguments were made for treating it either as a material entity or an immaterial kind of social construct related to other legal entities. The latter are not currently well described in BFO. Our solution is to define an *organization* by things that are true about it: an *organization* is a *continuant* entity that can bear roles and has members. Members of organizations are either organizations in themselves or individual *human beings*. Members can play organization-specific member roles. This definition is broad enough to include for example a *regulatory agency* like the *U*.*S*. *Food and Drug Administration*.

### Applications of OBI

OBI is in active use by multiple projects and the following section give a series of examples to document the diverse applications enabled by OBI.

#### Adding semantic expressivity to data stored in the IEDB

The Immune Epitope Database (IEDB)[28] catalogs experiments that characterize the location and function of immune epitopes in infectious agents, allergens, transplants and auto-antigens. Information is entered into the IEDB through author submissions and through manual curation of the scientific literature. Authors submit data to the IEDB because they are contractually obligated by the National Institutes of Health (NIH) to do so or in some cases authors have requested submission of large datasets that are not very useful when published as unstructured supplemental material. Over 1,000,000 experiments have been entered into the IEDB to date and manual curation has covered more than 98% of all journal articles containing epitope information from infectious agents, allergens, transplants and auto-antigens. Like many databases, information in the IEDB comes mostly in the form of values from controlled lists. Where available, existing ontologies were used as a source for terms on such lists, but for many types of information controlled vocabularies had to be developed by the IEDB team. Building and maintaining controlled vocabularies that deal with changing naming conventions over decades of scientific practice was a significant task. Worse, it was a thankless task, as the work had limited value outside of the IEDB itself.

**Fig 3:**
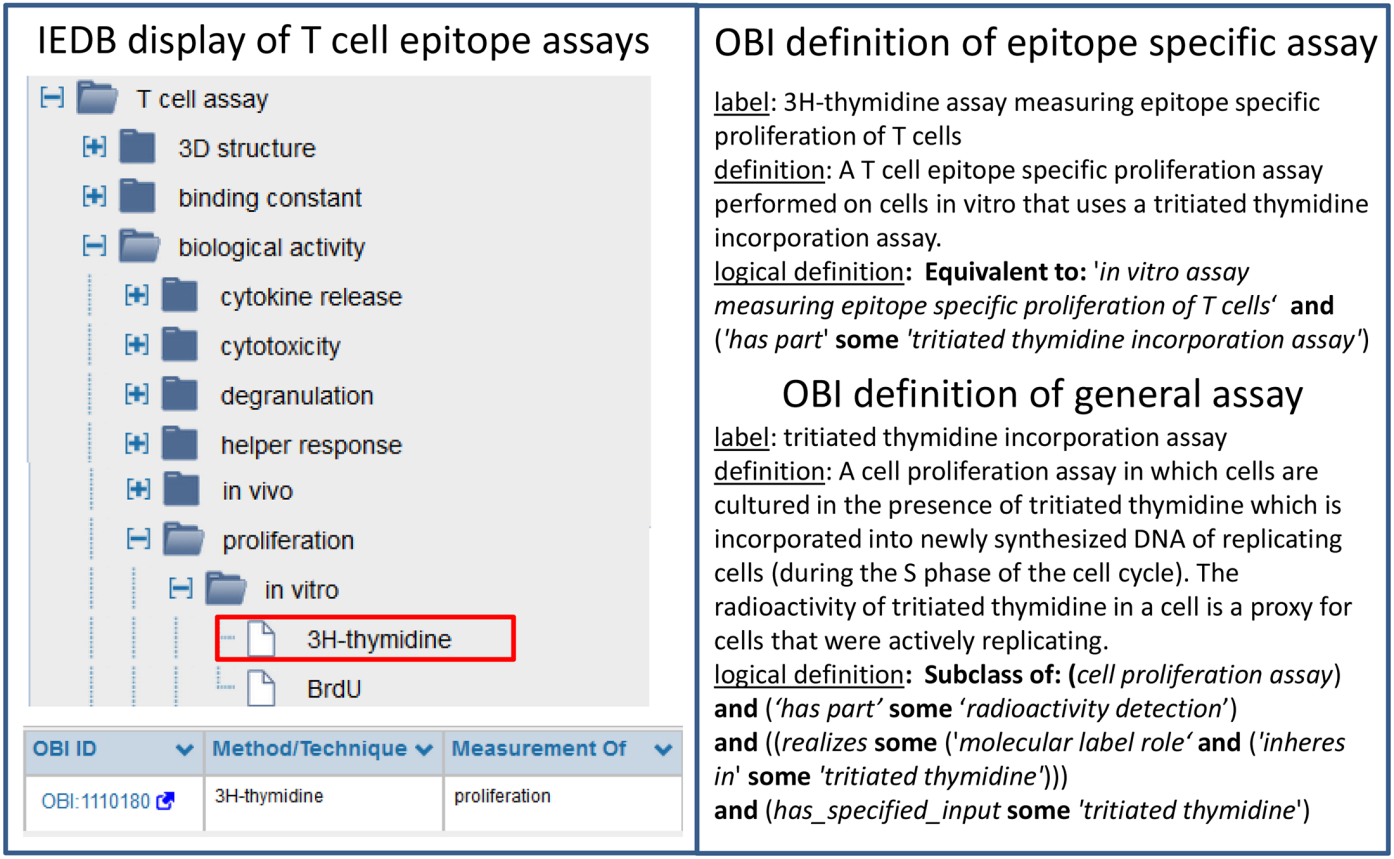
T cell epitope assays in the IEDB and OBI. The left hand panel illustrates how an IEDB user can select from different T cell epitope characterization assays in the IEDB. The labels utilized are shorthand which in the context of the assay tree in the IEDB is sufficient for an immunologist user to understand what assays are being denoted. Each assay in the IEDB refers to a formal definition in OBI (right hand panel). While the IEDB only captures with assays in which epitope specific proliferation is measured, the type of assay utilized (in this example thymidine incorporation) is applied in many other studies and is more likely to be re-usable.

To address this issue, the IEDB team has worked with multiple ontology developers to extend e.g. GO, ChEBI, Protein Ontology (PRO) and PATO to replace IEDB internal controlled vocabularies. By far the largest contribution was made working with OBI, which not only covers terms specific for experiments and investigations, but also provides the framework that explains how terms from other ontologies are related to each other in the context of an experiment. Fig 3 depicts how the list of T cell assay types used in the IEDB has been mapped to OBI. The T cell assay classes in OBI are constructed using logical definitions that tie them to GO terms representing the biological processes interrogated by the assays such as *IFN-gamma production* and *cell proliferation*, and to more general experimental techniques represented in OBI such as Enzyme-linked immunosorbent assay (ELISA) or Fluorescence-activated cell sorting (FACS) assays.

Using OBI as a source of assay terms in the IEDB has replaced plain lists of strings and given them expressive textual and logical definitions. This has multiple benefits in the maintenance of terms for the IEDB team. It is now easier to identify redundant entries as the use of GO terms provides synonyms to indicate that ‘CCL1 production’ is equivalent to ‘TCA3 production’. Logical reasoning also organizes terms into a hierarchy without the need for human intervention by identifying parent-child relationships between assay types that can be inferred based on parallel parent-child relationships in GO. For example, it is inferred that an *IFNg production assay* is a child of *cytokine production assay*. Such a hierarchy eases navigation of flat lists with hundreds of entries, and enables querying for more general terms such as ‘cytokine release assays’ or cell proliferation assays. The broader impact of this work with OBI is that these IEDB-motivated terms are now available to all.

#### Designing smart, standardized submission forms for EuPathDB

The Eukaryotic Pathogen Database (EuPathDB; http://eupathdb.org) project integrates genomic and functional genomics data from over 30 different protozoan parasite species. Protozoan parasites are a major cause of global human and veterinary infectious diseases, such as malaria, toxoplasmosis, cryptosporidiosis, Chagas disease, sleeping sickness and leishmaniasis. EuPathDB also aims to integrate data on specific isolates of parasites, their genotypes, and effects of genetic manipulation on the phenotype. However, currently available data on parasite isolates and genetic manipulation is highly heterogeneous and therefore would be hard to query and represent without community-accepted standards. EuPathDB is a National Institute of Allergy and Infectious Diseases (NIAID) Bioinformatics Resource Center (BRC) and has developed standards for this purpose with other BRCs and NIAID Genome Sequencing Centers for Infectious Disease [29]. OBI was used as the semantic basis for these standards to facilitate their creation and mapping to related standards such as the Genome Standards Consortium and to required fields for NCBIs BioSample/BioProjects checklists. The use of OBI as both a standard and common semantic reference also illustrates its broader impact.

To better standardize data as it is being captured, the EuPathDB team and user communities decided to develop submission forms. OBI was chosen as the basis for these forms because it provides a framework for modeling the generation of the desired data through its use of planned processes in which external ontologies can easily be referenced. In OBI, we describe the genotype of an isolate by first referring to the process of specimen collection that resulted in the physical isolate, followed by the sequencing experiment that was performed on the isolate, which provided the genotype information. We consulted with investigators performing these processes and established what was needed in the form. The result was a form that captures details about the process of creating an isolate specimen (where, when, and from what it was collected) and performing a sequencing assay (to obtain isolate sequence data). In this example, the terminology used for describing each instance of an isolate specimen is drawn mainly from other ontologies (e.g., Gazetteer (GAZ), PATO). OBI is used for categories of terms needed (e.g., sequence data) and for relating the information collected on the form for loading into a database and subsequent data mining. This approach has been used to create a form for collecting common data from the International Centers of Excellence for Malaria Research [30].

**Fig 4:**
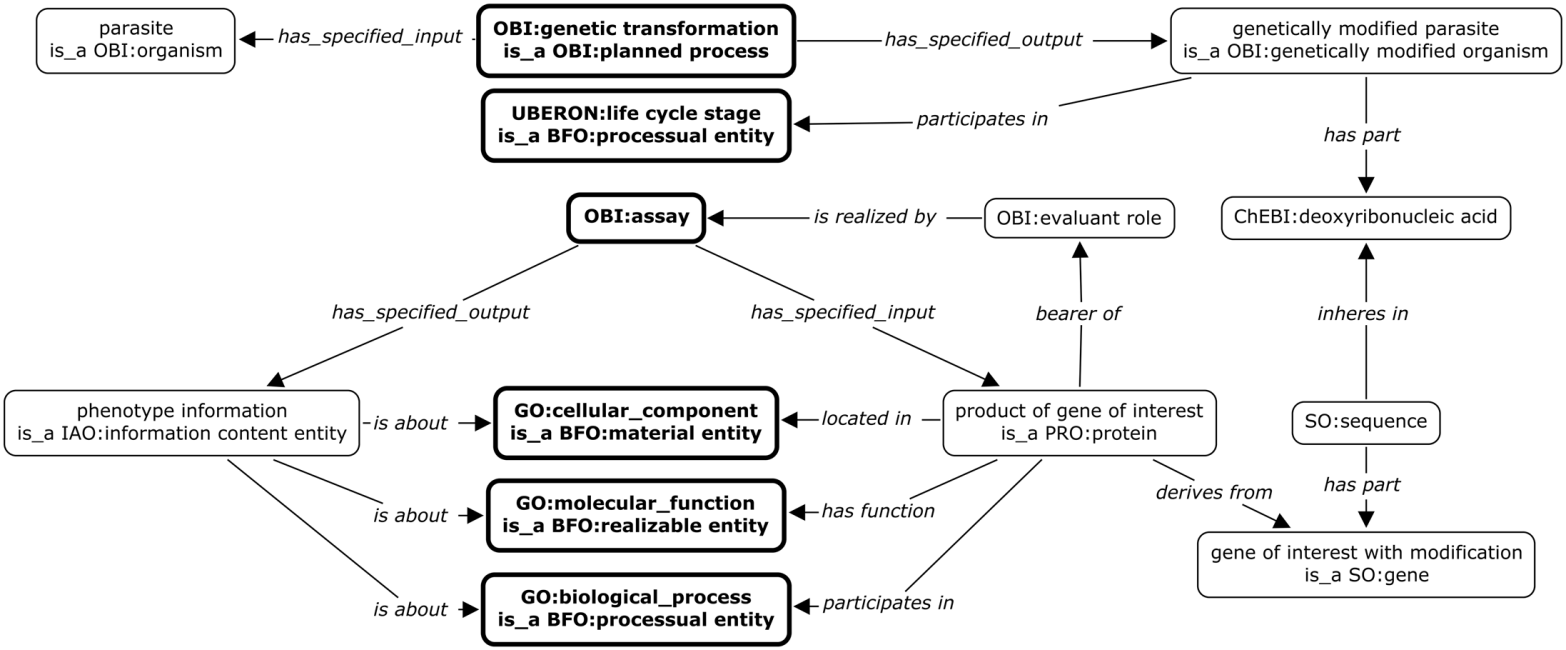
Ontology-based representation of phenotype data. The genetically modified parasite, a *genetically modified organism*, is generated by a *genetic transformation* process (top section). *Assays* are performed to examine the genetically modified parasite for the *cellular component* the gene product is located in, effects on its *molecular function*, or the *biological process* it participates in during a specific *lifecycle stage* (bottom section). The representation is at the instance levels, i.e. not all assays will have the specified inputs and outputs. The class mentions are to indicate what is being instantiated. Ontology terms are indicated by using ontology name abbreviations as prefix. Relations are italicized. The data collected in the submission form are in bold font. Fields requiring ontology terms are in thick border box.

The same approach is used for the more complex task of capturing phenotype information obtained by genetically modifying parasites. Insights into the function, location, and biological processes for parasite proteins of interest are found through genetic modification such as knocking out genes encoding the protein or tagging it with a fluorescent marker (Fig 4). Also of interest is the effect of these modifications on the viability, infectivity, and proliferative ability of the parasite. The key planned processes used to model this case are *genetic transformation* and *assay* (both drawn from OBI). In addition to using OBI terms to relate collected information for EuPathDB databases, they will also be used to populate the choices that users are presented with on a web-based form.

#### Harmonize the annotation across different functional genomics resources

The ISA project (short for Investigation, Study, Assay [31]) supports managing and tracking biological experiment metadata to ensure its preservation, discoverability and re-use, by providing a set of software applications centered around a user-friendly tabular data-entry format called ISA-Tab. ISA has been adopted by a variety of user groups in the ISA Commons [31]. This includes (i) international public repositories such as Metabolights [32] backing deposition of Metabolomics datasets, (ii) research consortia repositories (e.g. ToxBank [33], diXa (http://www.dixa-fp7.eu), (iii) standard development groups (e.g. ISA-Tab-Nano [34], COordination of Standards in MetabOlomicS (COSMOS) http://cosmos-fp7.eu) and, last but not least, (iv) data journals such as BMC BioMedCentral GigaScience [35] and Nature Publishing Group Scientific Data (http://www.nature.com/sdata/).

The ISA model is supported by a set of software applications to configure annotation requirements and create ISA tables, load information into databases and convert ISA representations into a range of formats for deposition to public repositories (e.g. The European nucleotide archive (ENA) [36], ArrayExpress [37]). The workflow of the ISA system has been validated and is compatible with existing technology centric formats (e.g. MAGE-TAB for ArrayExpress [37] and Sequence Read Archive (SRA) EXtensible Markup Language (XML) for ENA [36], respectively). It can be applied to import data into analysis environments or publish experimental metadata alongside a narrative article [38–41].

**Fig 5:**
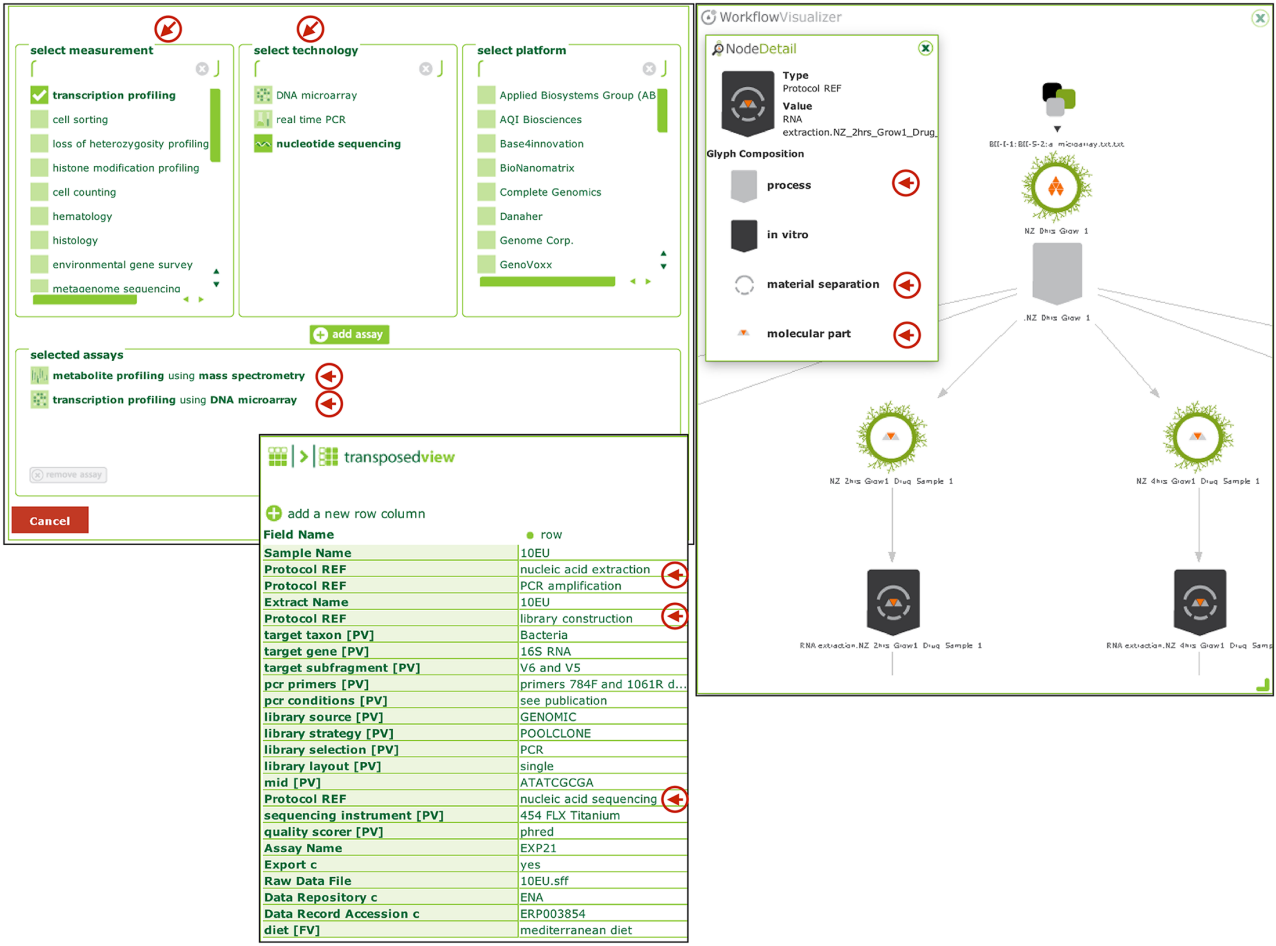
Panel (a) shows the assay selection panel in ISAcreator (editor and curation tool) which uses OBI terms. Panel (b) shows ISA ‘Protocol REF’ elements, which are annotated with subclasses of ‘OBI:planned process’. Panel (c) shows a glyph-based representation of the underlying mapping of ISA syntax element into OBI framework: circles correspond to Material or Data, which are ‘specified_input_of’ some ‘OBI:planned process’ either ‘OBI:material transformation’ or ‘OBI:data transformation’. The graph matches by the underlying RDF representation.

ISA-Tab syntax encourages the use of controlled terminology whenever possible. OBI is the recommended source of vocabulary to define ISA Assay types via the 'Technology' and 'Measurement' type pairs, as well as to annotate ISA Protocol Type attribute. OBI thus provides key classes whose instances are collected using ISA format. The default ISA configurations (templates for creating ISA-Tab files) provided with the tools rely on OBI terms (Fig 5 panel a and b).

Finally, the latest ISA infrastructure software component, linkedISA [42] delivers an RDF conversion capability, rendering ISA experimental descriptions as linked data. The conversion engine uses one or more mappings between ISA syntactic elements and ontologies, and it can exploit OWL constructs. The primary mapping was devised to rely on a set of BFO-based ontological artefacts, with OBI as the main provider, thus defining articulated, coherent and traceable curatorial framework. The OBI-based RDF conversion enables automated semantic validation and annotation enhancement, for instance by inferring study design types following inspection of the experimental graph (Fig 5 panel c). Queries such as ‘retrieve all studies with balanced design’ or ‘retrieve all studies where study groups have at least 3 samples’ are now possible without any increase of submitters’ annotation burden [40]. Generic RDF serialization ensures conversion of ISA assays based on standard patterns. Refinements and specialization can be added by supplying a new mapping between domain specific ISA configurations and OBI representations, thus granting more precision. This extensibility means that representations can improve as OBI and ISA continue to develop. An example of ISA extensions to targeted metagenomics application and mapping to OBI and OBO Foundry resources has been discussed in a detail [43]. This extensibility to other communities provides an additional illustration of the broader impact of OBI adoption.

## Discussion

OBI is a community-driven ontology that represents biomedical investigations in a computationally amenable form. It enables the representation of investigations across disparate biomedical disciplines and is capable of doing so in a rich, precise and explicit way. As such it is a substantial cross-community attempt to support the annotation of the experimental context of biomedical data.

OBI fits in a broader framework. Since the Gene Ontology demonstrated the utility of a controlled, shared and explicit description of gene function, there has been a substantial growth in efforts to describe knowledge in biology. OBI integrates supports and extends many of these efforts including the formation of an Information Artifact Ontology. It provides a vocabulary for use with minimum information standards [3]. It complies with, and has helped to drive, the OBO Foundry principles [10, 11], and is a reviewed member ontology of the OBO Foundry. As more data represented using OBI become available, as exemplified by the provided use cases, its utility to the broader biomedical community will continue to increase.

Covering all biomedical investigation gives OBI a broad scope. This is important and necessary if it is to enable data integration across biomedicine. It is unlikely that it would have been possible to address a scope of this breadth without the wider ontology community. Although clearly necessary for biomedical investigations, we have not had to describe, for example, taxonomy, anatomy or genetics; rather, we have reused existing ontologies and have developed the MIREOT specification to support this.

Despite this, the breadth of OBI’s scope still poses a significant challenge. In order to garner the experience and knowledge to describe biomedicine, we have taken a community-based approach. Authors on this paper, for example, cover many disciplines within biomedicine. In itself, this community approach presents a substantial challenge; it is hard to build consensus, both of terminology and meaning, among individuals from divergent backgrounds. We have addressed this by focusing, firstly, on assigning a clear, precise and unambiguous meaning for each class in the ontology using both the logical and natural language definitions. Secondly, where terminology in sub-disciplines fails to make distinctions in meaning or is inconsistent between communities, we have addressed this with the liberal addition of synonyms and with examples of usage for every class within the ontology.

While reuse of other ontologies has been critical to OBI, it also creates challenges. External ontologies are often large and are subject to change with independent release policies. Importing such large ontologies impacts on the scalability of the tools; independence may require consequent changes in OBI. We have addressed these issues with the development of the MIREOT mechanism which enables partial import of external ontologies[19]. Despite this, the size of the resulting ontology is significant and this has identified some shortcomings in the tooling. While Protégé-OWL has worked well as an editing tool, it requires expert knowledge for use with an ontology of this complexity. In particular, integration with versioning systems, essential for distributed development, is currently limited. Similarly, OWL has enabled computational reasoning, which is valuable for both use and development of the ontology. However, current reasoning tools do not provide a transparent explanation and debugging facility; likewise, performance is unpredictable and can change significantly between versions of OBI. The community continues to improve these tools and OBI has benefited from communication with their developers.

There have been prior efforts to generate a standardized representation of investigations. For example the Microarray Gene Expression Data (MGED) Ontology (MO) [44] was created to provide a standard terminology for microarray experiments. The developers of MO recognized that it was limited both in scope and by its structure [45]. Rather than create a new version of MO, effort was put into creating a Functional Genomics Ontology (FuGO) [46] with participants covering multiple technologies (e.g., microarray, mass spectrometry, flow cytometry) and multiple research areas (e.g., transcriptomics, genomics, metabolomics). It did not take long before it was recognized that as the effort to build FuGO grew, the participants were not limited to functional genomics and that much could be learned from independent efforts such as Ontology of scientific experiments (EXPO)[47]. The FuGO effort was rechristened the Ontology for Biomedical Investigations adjusting the scope to biological and medical investigations. OBI leveraged the use cases and terms provided by the prior efforts. Upper level ontologies were evaluated such as BFO[16], Descriptive Ontology for Linguistic and Cognitive Engineering (DOLCE) [48], and Suggested Upper Merged Ontology (SUMO)[49] to put the collected terms in an established framework. While each had its strengths and weaknesses, BFO was chosen due to its association with the nascent OBO Foundry [10] that would enable interoperability with other terminologies (e.g., Gene Ontology) needed to describe investigations.

OBI is complex, but then so are ‘materials and methods’ sections as they stand. There are ways of masking this complexity from end users, if required. One example is the Investigation, Study, Assay (ISA) infrastructure for data sharing [4, 50], which provides tools to configure and complete reports for specific types of biomedical investigations. The tools can set the minimal fields[3] required and restrict each of them to appropriate terms from OBI or other OBO ontologies without requiring the end user to have in-depth understanding of them. Similarly, we wish to ease the submission of new terms from the community to OBI. One method developed for this purpose utilizes template spreadsheets to create composite terms according to pre-defined design patterns[51]. We expect that more such tools will be developed that take full advantage of OBI internally, while providing an end user friendly interface.

Just as in science in general, new insights and findings will result in refinements and additions to OBI. As new terms get added, it is often necessary to also modify the definitions of existing terms to clearly delineate their scope. To ensure that such modifications are made consistently, OBI developers regularly review all terms in a subject area to ensure that definitions are distinct and to identify and enforce common design patterns. The next subject area for review will be the assay terms in OBI, for which over the past years, a large number of descendent terms have accumulated, resulting in a need to review the different design patterns, especially the logical definitions, to achieve better consistency. Assay terms described in the present paper may well have modified definitions in the upcoming months. Another driver of upcoming changes in OBI is the collaborative work with the Evidence Ontology (ECO) [52] which aims to utilize OBI to explicitly define how its widely used ‘evidence codes’ are related to specific experiments. Thus, the present paper provides a description of OBI as it is implemented right now, but users should always refer to the most current version of the ontology itself to ensure that it is used accurately. Changes to the definition of a term that leave the intended meaning intact will keep the term identifier intact. As this is true for the vast majority of changes, users will be alerted to major changes in the ontology for terms the use based on the term identifiers being deleted. However, the core of OBI, which has been recently reviewed internally by the OBI team, is considered stable with regards to the asserted hierarchy. Future changes will largely extend the power of what can be done with OBI through additional terms, axioms, and design patterns.

In summary, OBI represents a substantial and significant effort to describe biomedical investigations in a consensual, wide-ranging and computationally amenable way. Ontologies such as OBI are essential to rise to the challenges of large-scale biology, safeguard the inheritance of experimental data for the future, and maximize its usefulness in the present.

## Supporting Information

S1 Table Projects utilizing OBI. (XLSX)

## Abbreviations

API: Application Program Interface
BFO: Basic Formal Ontology
BRC: Bioinformatics Resource Center
CARO: Common Anatomy Reference Ontology
CC-by: Creative Commons Attribution
ChEBI: Chemical Entities of Biological Interest
COSMOS: COordination of Standards in MetabOlomicS
DL: Description Logic
DOLCE: Descriptive Ontology for Linguistic and Cognitive Engineering
ECO: Evidence Ontology
ELISA: Enzyme-linked immunosorbent assay
ENA: The European nucleotide archive
EuPathDB: Eukaryotic Pathogen Database
EXPO: Ontology of scientific experiments
FACS: Fluorescence-activated cell sorting
FuGE: Functional Genomics Experiment
FuGO: Functional Genomics Ontology
GAZ: Gazetteer
GO: Gene Ontology
IAO: Information Artifact Ontology
IEDB: Immune Epitope Database
IRI: Internationalized Resource Identifier
ISA: Investigation, Study, Assay
ISA-Tab: Investigation, Study, Assay tab-delimited format
MAGE-TAB: Microarray Gene Expression tab-delimited format
MGED: Microarray Gene Expression Data
MIREOT: Minimum information to reference an external ontology term
MO: Microarray Gene Expression Data Ontology
NCBI: National Center for Biotechnology Information
NCBO: National Center for Biomedical Ontology
NIAID: National Institute of Allergy and Infectious Diseases
NIH: National Institutes of Health
OBI: Ontology for Biomedical Investigations
OBO: Open Biological and Biomedical Ontologies, Open Biomedical Ontology
OWL: Web Ontology Language
PATO: Phenotype Attribute and Trait Ontology
PRO: Protein Ontology
RDF: Resource Description Framework
RDFS: Resource Description Framework Schema
RO: Relations Ontology
SRA: Sequence Read Archive
SPARQL: SPARQL Protocol and RDF Query Language
SQL: Structured Query Language
SUMO: Suggested Upper Merged Ontology
UBERON: Uber anatomy ontology
W3C: World Wide Web Consortium
XML: EXtensible Markup Language
